# Case Report: An extremely rare case of epithelioid STUMPs

**DOI:** 10.3389/fonc.2025.1702908

**Published:** 2026-01-07

**Authors:** Danjie Li, Yibei Guan, Ziqiu He, Yanru Wang, Xinying Liu, Miao Xiong, Min Min

**Affiliations:** 1 Department of Obstetrics and Gynecology, Shanghai Sixth People’s Hospital Affiliated to Shanghai Jiao Tong University School of Medicine, Shanghai, China; 2 Department of Pathology, Shanghai Sixth People’s Hospital Affiliated to Shanghai Jiao Tong University School of Medicine, Shanghai, China

**Keywords:** epithelioid leiomyomas, epithelioid STUMP, hysterectomy, malignant potential, STUMP

## Abstract

**Introduction:**

Uterine leiomyomas are the most common benign tumors of the female reproductive system. Epithelioid STUMP is a special and relatively rare type of uterine leiomyoma that may exhibit malignant potential as smooth muscle tumors with an epithelioid cell morphology are generally believed to be more aggressive than usual leiomyomas. This report describes a case of epithelioid STUMP.

**Case description:**

A 45-year-old woman of childbearing age underwent curettage for abnormal uterine bleeding. The initial pathological findings suggested a low-grade malignant uterine mesenchymal tumor; therefore, laparoscopic-assisted vaginal hysterectomy, bilateral adnexal resection, and retroperitoneal lymph node biopsy were performed. Postoperative pathology revealed small, round to short, spindle-shaped tumor cells of uniform size and mild pleomorphism, intermingled with smooth muscle of the uterine wall, with mitotic figures 2-3 per 10 high-power field, suggesting an extremely rare, epithelioid STUMP. The tumor exhibited infiltrative growth at its periphery, indicating malignant potential. The patient did not undergo radiotherapy or chemotherapy postoperatively and the last follow - up after surgery showed that the patient had not recurred for 8 months.

**Conclusion:**

Epithelioid STUMP, a specific subtype of uterine leiomyomas, may exhibit malignant potential. For this subtype, thorough preoperative examinations (enhanced pelvic magnetic resonance imaging and enhanced abdominal computed tomography) are necessary to avoid misdiagnosis or delayed diagnosis, which can worsen the patient’s condition, and to establish appropriate diagnostic, therapeutic, and prognostic strategies.

## 1 Introduction

Uterine leiomyomas are the most common benign tumors of the female reproductive tract, primarily affecting women aged 30–50 years. Statistics indicate that at least 89% of all women of reproductive age have uterine fibroids. However, as most patients lack or do not exhibit clinical symptoms, the actual incidence rate is likely higher (1–3). According to the 2020 edition of the World Health Organization classification of female reproductive tract tumors, the morphological subtypes, of uterine leiomyomas include cell-rich leiomyomas, apopletic leiomyomas, leiomyomas with bizarre nuclei, mitotically active leiomyomas, fumarate hydratase-deficient leiomyomas, fatty leiomyomas, edematous leiomyomas, mucinous leiomyomas, epithelioid leiomyomas (ELs), and (lobular) segmented leiomyomas (4, 5).

EL is a special type of uterine leiomyoma that was first described in 1965 and is relatively rare in clinical practice (6). Although the prognostic factors of epithelioid uterine leiomyomas are not yet clear, epithelioid cell-type smooth muscle tumors are believed to be more dangerous than usual leiomyomas. Patients with EL often lack specific clinical manifestations and are clinically rare, making misdiagnoses and missed diagnoses common, particularly when distinguishing EL from low-grade malignant endometrial stromal sarcomas (6–8).

Epithelioid uterine smooth muscle tumors of uncertain malignant potential(STUMPs) are tumors exceeding the criteria for EL (rounded or polygonal cells with eosinophilic granular or clear cytoplasm lacking cytologic atypia, < 0.8 mitoses/mm2 or < 2 mitoses/10 HPF, FD = 0.55, 0.24 mm2 in area) but falling short of a diagnosis of epithelioid leiomyosarcoma (moderate to severe cytologic atypia or coagulative necrosis or ≥ 1.6 mitoses/mm2 or ≥ 4 mitoses/10 HPF, FD = 0.55, 0.24 mm2 in area) (4).In the absence of coagulative tumor necrosis and atypia, EL with mitotic counts of 2–3 per 10 high-power fields (HPF) is classified as epithelioid STUMP (4, 7).

Here, we discuss a case of uterine fibroids, which is rare in terms of pelvic magnetic resonance imaging and pathological diagnosis, that was eventually diagnosed as rare, epithelioid STUMP.

## 2 Case description

A 45-year-old female patient, G4P2, of reproductive age with regular menstrual cycles presented to Anqiu People’s Hospital with the chief complaint of intermittent vaginal bleeding for over 2 months. The patient without medical and family history underwent a hysteroscopic diagnostic curettage. The pathology report indicated a suspected low-grade malignant uterine stromal tumor, with a recommendation for referral to a specialist hospital. Immunohistochemistry of the curettage specimen revealed the following: wild-type p53, positive ER (high intensity 80%), PR (high intensity 80%), desmin, and SMA, weakly positive CK, focal and weakly positive EMA, and negative CD10 and CD117. Ki67 was 2%. Two days later, the patient was admitted to our hospital for treatment. Upon admission, laboratory blood tests were performed, and the relevant finding included a hemoglobin level of 98 g/L (**Table 1**).

**Table 1:** Blood test results and main IHE markers of the patient done before surgery.

Test	Result
Hemoglobin	98g/L
Beta hCG	negative
AMH	0.127ng/dl
Estradiol	78.4pg/ml
FSH	42pg/ml
Serum Markers	
CA125	negative
LDH	negative
Main IHE Markers	
ER	strong positive
PR	strong positive
Desmin	positive
SMA	positive
Ki-67	positive

## 3 Diagnostic assessment

**Figure 1:**
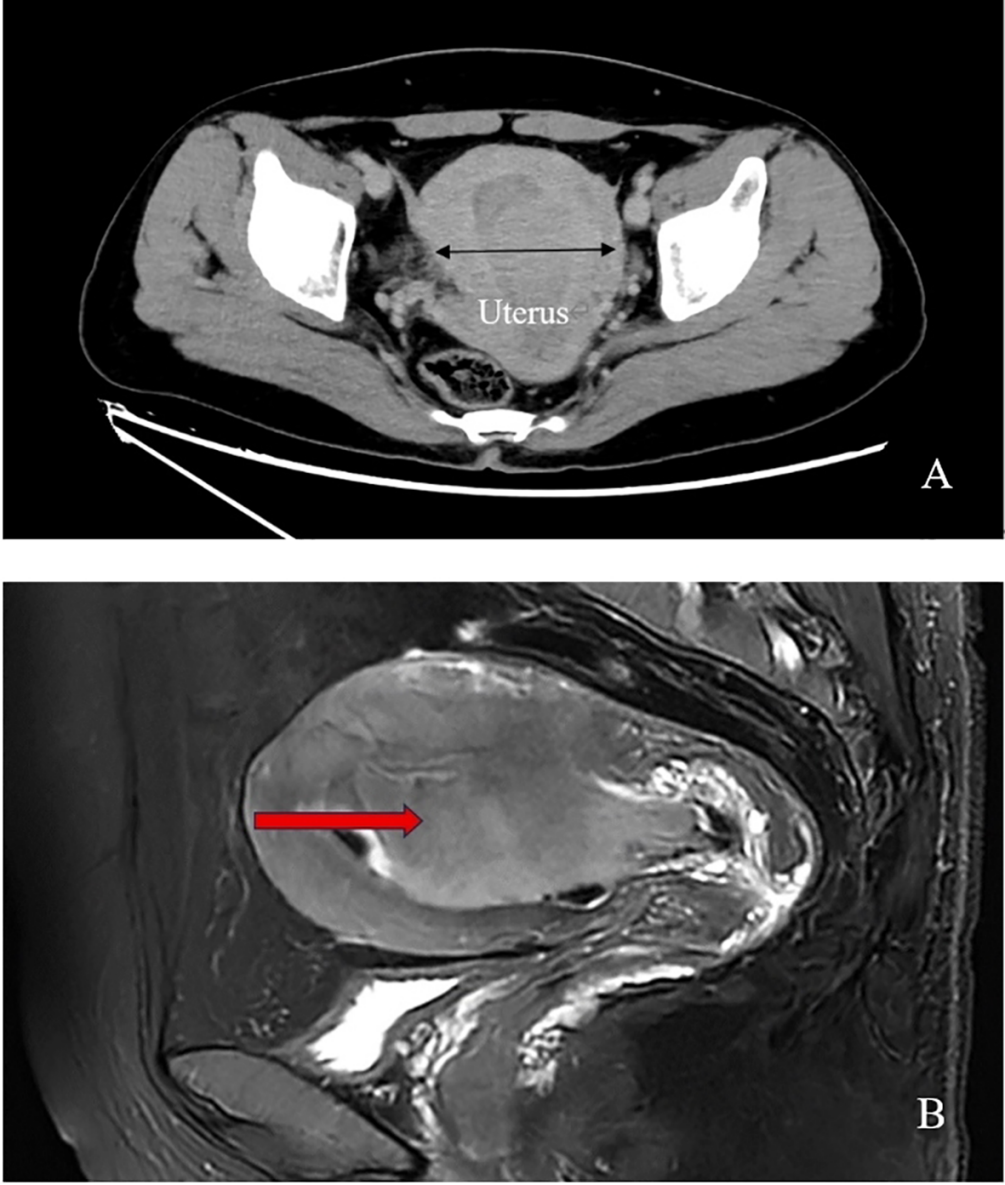
**(A)** Pelvic enhanced computed tomography scan of the patient shows increased uterine size and a protruding oval-shaped mass in the uterine cavity; **(B)** Pelvic magnetic resonance imaging reveals an anteverted and bulky uterus with a large oval-shaped mass (arrow point) occupying the entire uterine cavity, resulting in blur endothelium.

**Figure 2:**
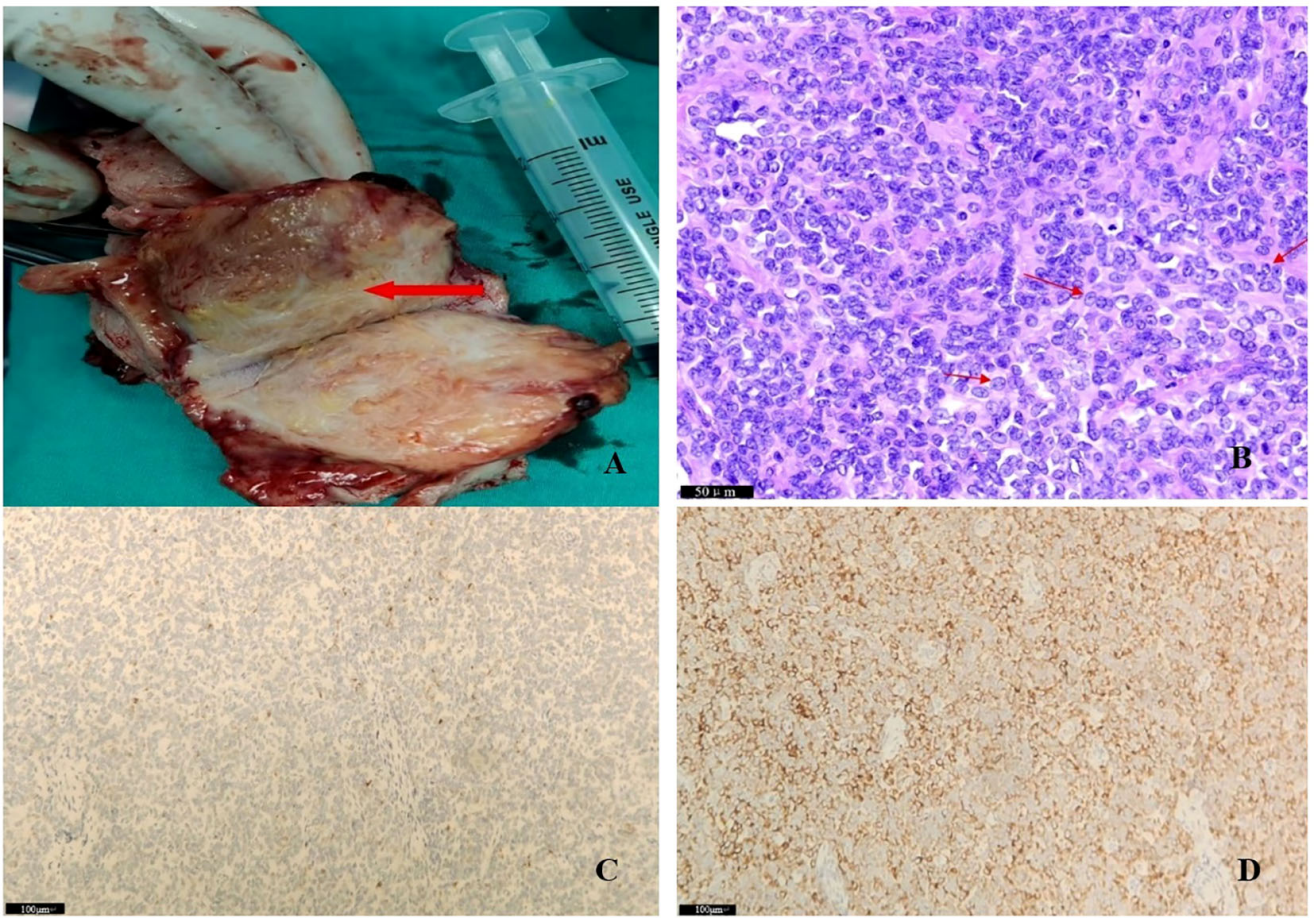
**(A)** Macroscopic image of the resected uterine specimen during surgery; **(B)** Final pathology: filled with atypia epitheloid cells (see arrows); areas with low mitotic activity (2-3/10 high power fields) were noted; **(C)** pancytokeratin positive; **(D)** desmin positive.

**Figure 3:**
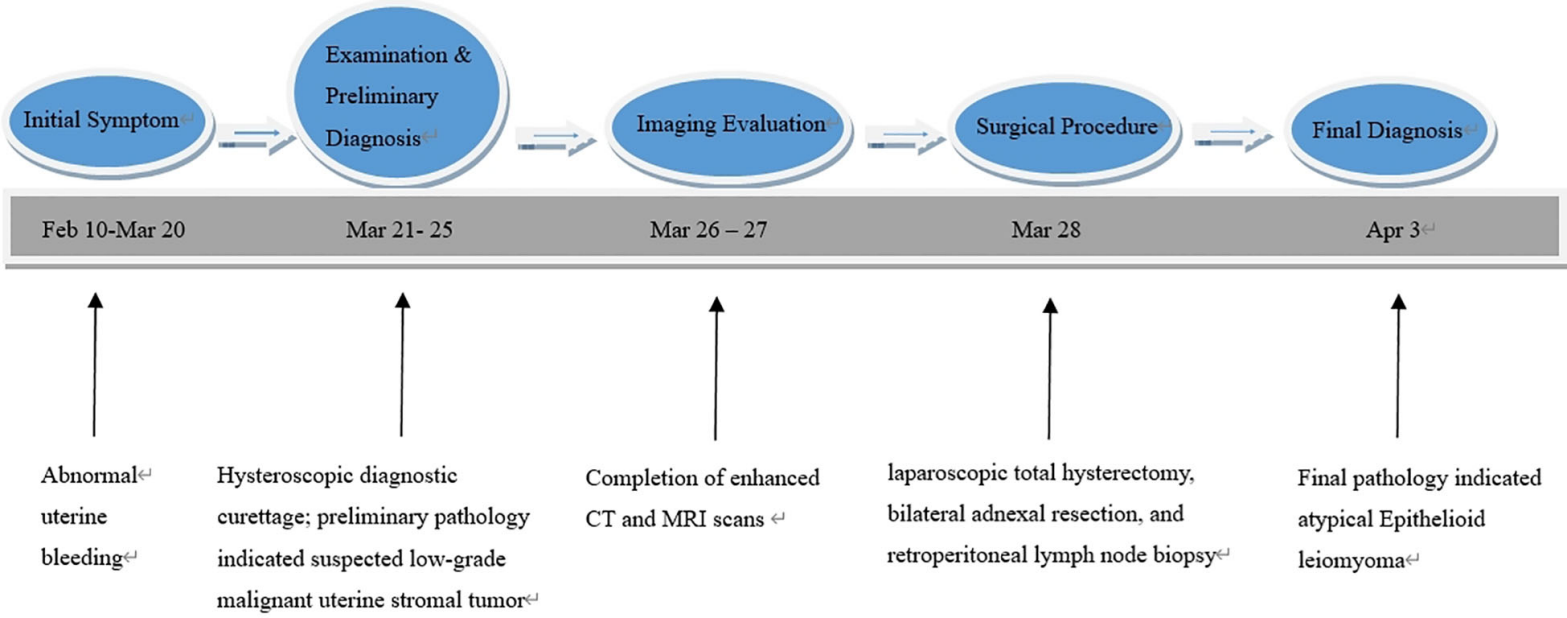
Timeline of the patient’s disease and treatment course.

Enhanced computed tomography scan of the lower abdomen showed enlarged uterine volume, marked thickening of the left uterine wall, an oval-shaped mass protruding from the uterine cavity with a maximum cross-sectional dimension of approximately 36 × 71 mm, marked enhancement after contrast administration, normal bladder filling, no obvious enlarged pelvic lymph nodes, and no obvious pelvic effusion (**Figure 1A**). The examination mentioned above showed an enlarged uterine volume, marked thickening of the left uterine wall, and an elliptical mass protruding from the uterine cavity, suggesting a possible sarcoma. Pelvic magnetic resonance imaging with contrast showed an elliptical abnormal signal focus measuring approximately 42.7 × 71.5 mm in the uterine cavity, appearing as low signal on T1-weighted images and slightly higher signal on T2-weighted images, with restricted diffusion. The lesion had unclear boundaries with the left posterior uterine wall muscle layer and showed significant enhancement after contrast administration (**Figure 1B**). A scar shadow was seen on the anterior wall of the lower uterine segment; multiple Nabothian cysts were observed in the cervix; there was a small amount of blood in the uterine cavity; and no obvious enlarged lymph node or fluid accumulation was seen in the pelvis. No obvious abnormal enhancement foci were observed after contrast administration. Based on these findings, the possibility of a uterine endometrial stromal sarcoma was suspected. Concurrent pathological assessment showed small, round to short, spindle-shaped tumor cells with infiltrative growth in the endometrial layer and myometrium of the uterus. The cells were of uniform size and had a mild pleomorphism, with mitotic figures <5 per 10 high-power fields (HPF). Based on the immunohistochemical findings, the diagnosis was suspected to be a mesenchymal tumor of intermediate or low malignancy grade. Therefore, close follow-up was recommended. Immunohistochemistry results showed positive pan-cytokeratin and desmin. CD34, H-caldesmon, HMB45, MyoD1, myogenin, SF-1, and SALL4 were negative. Ki67 was 5%. Based on the pathological assessment, it was difficult to definitively categorize the tumor as benign or malignant; however, potential for malignant transformation was suggested. Considering the patient’s age, marital status, reproductive history, and risk of malignancy, and excluding contraindications for surgery, laparoscopic total hysterectomy, bilateral adnexal resection, and retroperitoneal lymph node biopsy were performed. During the procedure, the uterus was enlarged to the size of a 3-month gravid uterus; bilateral ovaries and fallopian tubes appeared normal. However, there were partial adhesions between the bilateral fallopian tubes, ovaries, uterine wall, and intestinal tract. During the procedure, the uterus was incised, and a protruding, hard mass measuring approximately 6×4 cm was observed within the uterine cavity (**Figure 2A**). The surgery was performed smoothly, and approximately 300 mL of blood was lost during the procedure. The patient recovered well after surgery. Postoperative pathological assessment of the resected uterus and bilateral adnexa showed small, round to short, spindle-shaped tumor cells intermingled with the smooth muscle of the uterine wall. The cells were uniform in size and had mild pleomorphism, with mitotic figures 2-3 per 10 HPF (**Figure 2B**). Immunohistochemistry indicated the following findings: the tumor cells were positive for pancytokeratin (**Figure 2C**), vimentin, desmin(**Figure 2D**), and calretinin and weakly positive for WT-1, D2-40, CK5/6, and ALK. HMB45, h-caldesmon, MyoD1, myogenin, INSM-1, CD56, CD34, ERG, cyclin D1, SF-1, and SALL4 were negative; Ki67 was 5%. Based on the previous curettage biopsy and postoperative immunohistochemical results, the patient was diagnosed with epithelioid STUMP. The tumor exhibited infiltrative growth at its periphery, indicating malignant potential. Therefore, a close follow-up was recommended. The resected specimen also showed endometrial hyperplasia, chronic cervicitis, Nabothian cysts, multiple follicular cysts in both ovaries, and paramesonephric duct cysts. However, as the final pathology did not indicate a malignant tumor, no adjuvant therapy, such as radiotherapy or chemotherapy, was administered. The patient was discharged smoothly on the 7th day post-surgery. The last follow - up after surgery showed that the patient had not recurred for 8 months. Timeline of the patient’s disease and treatment course was shown in **Figure 3**. CARE Checklist of information was summarized in the **Supplementary Data Sheet 1**.

## 4 Discussion

Uterine leiomyomas are the most common gynecological tumors in women of reproductive age and typically have well-defined diagnostic criteria, prognoses, and standardized treatment protocols. However, in addition to typical benign leiomyomas and malignant leiomyosarcomas, several other tumor variants with distinct histomorphological and clinical-behavioral characteristics have been identified (9, 10). Uterine smooth muscle tumors of uncertain malignant potential (STUMP), a distinct subtype of uterine smooth muscle tumors, are often misdiagnosed as malignant uterine leiomyosarcomas due to the absence of typical clinical manifestations and effective preoperative diagnostic tools (11). Its final diagnosis can only be confirmed by pathological report. Several researchers have proposed pathological diagnostic criteria for STUMP, and the histopathological criteria proposed by Gupta in 2018 have been widely adopted in China. Gupta et al. suggest that STUMP may be diagnosed when two or more of the following criteria are met: 1) tumor necrosis is unclear and difficult to classify; 2) the tumor exhibits diffuse or multifocal atypia, with a mitotic index of 8–9/10 HPFs at the tumor margin, or the lesion shows diffuse atypia; 3) tumor cells have a mitotic index >15/10 HPFs without cellular atypia or necrosis; 4) the tumor exhibits irregular or multifocal ischemic necrosis or coagulative necrosis; 5) the tumor presents with mucinous and epithelial-like morphology, showing atypia or active proliferation; 6) peripheral muscle layer invasion is present, but other malignant tumor histological features are absent; and 7) the presence of atypical mitotic figures, but without other histological features of malignant tumors (12, 13).

STUMP generally grows slowly, with low rates of recurrence and metastasis, and a 5-year overall survival rate of 90–100% (14). Even among patients who require further treatment after recurrence, most achieve long-term survival. The recurrence rate for STUMP ranges 7%–28%, depending on the criteria used. The epithelial and mucinous subtypes may be more prone to recurrence. The duration before recurrence is longer than that of leiomyosarcoma (average, 47 months), and recurrent tumors may appear histologically similar to the original tumor or may transform to leiomyosarcoma. The median survival time for patients with recurrence is 5.5 years (15). Although immunohistochemical markers such as p16, p53, Ki-67, p21, BCL2, ER, and PR have been used to assist in the diagnosis and prediction of prognosis, the results have been unsatisfactory. Genomic index greater than 35, amplification of chromosomes 5p and 17p, and deletion of chromosome 13 are considered poor prognostic factors (16).

Given that STUMP is typically diagnosed through postoperative pathological examination, it is essential to consider the patient’s age and reproductive status when determining treatment strategies for patients who have undergone myomectomy (17–20). A literature review reported that among 67 patients with STUMP who underwent myomectomy, 5 (6.6%) experienced recurrence, and 1 of these 4 patients died from recurrence 1 year after the initial surgery (21). Given its low recurrence rate and typically late onset, fertility-sparing surgery is considered safe and feasible if the patient is of reproductive age and desires fertility preservation (22). However, patients should be provided with a detailed explanation of the unpredictable nature of the disease, and informed consent should be obtained following consultation. For patients who have completed childbearing or have no fertility requirements, a total hysterectomy with or without bilateral salpingo-oophorectomy should be performed to reduce the risk of recurrence. Currently, no published reports indicate that postoperative adjuvant hormone therapy or chemotherapy can reduce the recurrence rate of this disease.

In this case, as the patient was nearing menopause and had no desire for future pregnancies, a total hysterectomy was recommended to prevent residual tumor tissue after surgery. The tumor measured 4–6 cm in size with uniformly sized cells and a benign morphology; thus, no adjuvant radiotherapy or chemotherapy was administered. While the reported mitotic score was 2–3 mitotic figures per 10 HPF, the tumor exhibited infiltrative growth at the periphery, indicating malignant potential, which met the definition of epithelioid STUMP. Regular outpatient follow-ups have been recommended to the patient to monitor for recurrence or metastasis.

## 5 Conclusion

Uterine leiomyomas are common pathological conditions of the uterus that can be confused with malignant tumors, especially in cases with abnormal growth patterns. Definitive treatment involves myomectomy and hysterectomy, followed by regular follow-up for prognosis. For epithelioid STUMP, thorough preoperative examinations are necessary to avoid misdiagnosis or delayed diagnosis and to establish appropriate diagnostic, therapeutic, and prognostic strategies.

## Patient perspective

The patient reported that the surgical procedure was tolerable, with minimal postoperative pain. She expressed gratitude for the improved mobility and reduced pain, which significantly enhanced her quality of life. She remains optimistic about her long-term recovery and is committed to adhering to the rehabilitation plan.

## Data Availability

The original contributions presented in the study are included in the article/**Supplementary Material**. Further inquiries can be directed to the corresponding authors.
